# Individualized embryo selection strategy developed by stacking machine learning model for better in vitro fertilization outcomes: an application study

**DOI:** 10.1186/s12958-021-00734-z

**Published:** 2021-04-05

**Authors:** Qingsong Xi, Qiyu Yang, Meng Wang, Bo Huang, Bo Zhang, Zhou Li, Shuai Liu, Liu Yang, Lixia Zhu, Lei Jin

**Affiliations:** Reproductive Medicine Center, Tongji Hospital, Tongji Medical College, Huazhong University of Science and Technology, No.1095, Jiefang Road, Wuhan, 430030 China

**Keywords:** Artificial intelligence, Embryo selection, Machine learning, In vitro fertilization, In vitro fertilization prediction

## Abstract

**Background:**

To minimize the rate of in vitro fertilization (IVF)- associated multiple-embryo gestation, significant efforts have been made. Previous studies related to machine learning in IVF mainly focused on selecting the top-quality embryos to improve outcomes, however, in patients with sub-optimal prognosis or with medium- or inferior-quality embryos, the selection between SET and DET could be perplexing.

**Methods:**

This was an application study including 9211 patients with 10,076 embryos treated during 2016 to 2018, in Tongji Hospital, Wuhan, China. A hierarchical model was established using the machine learning system XGBoost, to learn embryo implantation potential and the impact of double embryos transfer (DET) simultaneously. The performance of the model was evaluated with the AUC of the ROC curve. Multiple regression analyses were also conducted on the 19 selected features to demonstrate the differences between feature importance for prediction and statistical relationship with outcomes.

**Results:**

For a single embryo transfer (SET) pregnancy, the following variables remained significant: age, attempts at IVF, estradiol level on hCG day, and endometrial thickness. For DET pregnancy, age, attempts at IVF, endometrial thickness, and the newly added P1 + P2 remained significant. For DET twin risk, age, attempts at IVF, 2PN/ MII, and P1 × P2 remained significant. The algorithm was repeated 30 times, and averaged AUC of 0.7945, 0.8385, and 0.7229 were achieved for SET pregnancy, DET pregnancy, and DET twin risk, respectively. The trend of predictive and observed rates both in pregnancy and twin risk was basically identical. XGBoost outperformed the other two algorithms: logistic regression and classification and regression tree.

**Conclusion:**

Artificial intelligence based on determinant-weighting analysis could offer an individualized embryo selection strategy for any given patient, and predict clinical pregnancy rate and twin risk, therefore optimizing clinical outcomes.

## Introduction

For decades, discussions about how to improve the clinical outcomes of in vitro fertilization (IVF) treatment have persisted. Multiple-embryo transfer was suggested to increase the possibility of successful implantation but inevitably elevated the risk of multiple-embryo gestation. IVF-associated multiple pregnancies exhibit significant financial, social, and medical implications [1, 2]. Even though the transfer of embryos has been limited to no more than two in recent years, the overall twin rate worldwide after assisted reproduction has still varied from 15 to 30% [3]. The incidences of premature birth, low birth weight, cerebral palsy, neurological complications, and perinatal mortality of twin pregnancy markedly increased compared with singleton pregnancy [4]. To minimize the rate of multiple-embryo gestation, significant efforts, including individualized service provision and single embryo transfer (SET) enhancement, have been made in the course of these decades.

Embryo morphological analysis is the routine method for selecting the highest-quality embryos to transfer. It is commonly suggested to perform SET on prognostically good patients with a top-quality embryo and to perform double embryo transfer (DET) on prognostically poor patients in whom good-quality embryo is unavailable in the IVF lab. In a large proportion of IVF patients with sub-optimal prognosis or with medium- or inferior-quality embryos, the selection between SET and DET could be perplexing. Because many features have been shown to influence embryo implantation potential positively or negatively in IVF [5–8], developing an optimal embryo selection plan to balance maximum clinical pregnancy rate and minimum twin risk is complicated for IVF clinicians. Therefore, a more precise, quantified, stable embryo selection model based on all possible influencing determinants needs to be constructed. This will potentially offer evidence-based patient counseling and predictable successful chances for any given patient.

Artificial intelligence (AI) represents the combination of machine learning, and a moderation and self-adapting prediction model. Previous studies related to machine learning in IVF mainly focused on selecting the top-quality embryos to improve IVF outcomes [9–13]. Developing a flexible, individualized embryo selection approach based on available embryos of various qualities and different twin rate threshold settings is another promising issue.

The purpose of our study is to construct an individualized embryo selection strategy and pregnancy prediction model, developed by stacking machine learning, to identify features correlated with embryo implantation potential and to evaluate available embryos’ implantation chances quantitatively. We aim to balance maximal clinical pregnancy and minimal acceptable twin risk in IVF with this model and validate its clinical effectiveness and practicability in subsequent cycles.

## Materials and methods

### Study design and participants

IVF patients in Tongji Hospital between January 2016 and December 2018, with one or two embryo transfers in the fresh cycle, were enrolled in our study. Exclusion criteria included patients with (1) blastocyst transfer in fresh cycle; (2) oocyte donation cycles; (3) vitrified/warmed oocytes; (4) oocytes partially cryopreserved in fresh cycle; (5) combined vitrified/warmed embryo transfers. In total, 5828 patients, between January 2016 and March 2018 were included as the training set to construct our model. Thirty-eight features were analyzed in our study, and their baselines on training and validation sets were exhibited, among which 21 main variables were selected and listed in Table 1, including patient features, embryo morphology features, and embryo scores. After model construction, the developed embryo selection strategy was applied to guide the selection of SET or DET in 3383 individual cases between April and December 2018 as the validation set.

**Table 1:** Baseline characteristics of the variables included in the training and validation data sets

Features	Training set (***n*** = 5828)	Validation set (***n*** = 3383)
Patient composition		
SET	5264	3082
DET	564	301
Age*, y	30.46 ± 4.20	30.70 ± 3.94
Attempt times of IVF*	0.95 ± 0.43	0.78 ± 0.54
Antral follicle count*	13.64 ± 6.13	13.37 ± 6.36
Follicle stimulating hormone*, IU/L	7.59 ± 2.35	7.68 ± 2.79
Luteinizing hormone*, IU/L	4.71 ± 3.26	4.95 ± 6.08
E2 per mature oocyte, pmol/L	309.60 ± 141.26	265.68 ± 133.16
E2 on HCG day*, pmol/L	2810.88 ± 1424.43	2174.64 ± 1046.56
Endometrial Thickness*, mm	11.79 ± 2.40	11.98 ± 2.55
MetaphaseII(M II)*	9.86 ± 4.14	9.25 ± 4.02
2pronucleus(PN)*	6.79 ± 3.35	6.31 ± 3.22
Oocyte Number^a^*	11.03 ± 4.45	10.64 ± 4.49
2PN/MII*	0.70 ± 0.19	0.69 ± 0.20
Frozen Sperm	6.0%	6.3%
Male Factor^b^		
Oligospermia	9.2%	10.3%
Asthenospermia	12.8%	20.0%
Azoospermia	7.1%	9.1%
Female Factor^b^		
Endometriosis	3.0%	4.4%
Ovulation Disorder	5.9%	7.8%
Unknown	5.2%	12.3%
Sperm Retrieval		
Ejaculation	95.2%	95.2%
MESA	0.3%	0.7%
TESA	1.0%	1.3%
PESA	3.5%	2.8%
Stimulation Protocol^b^		
Agonist Protocol*	71.4%	70.8%
Antagonist Protocol	22.8%	28.5%
Endometrial Type^b^		
A*	83.3%	85.0%
B	2.0%	0.4%
C*	21.4%	19.7%
Infertility^c^		
Primary	64.9%	70.4%
Secondary*	35.1%	29.6%
Fertilization Method^b^		
IVF	72.5%	57.9%
ICSI*	24.5%	34.8%
Embryo Features		
Number of Blastomere*	7.93 ± 0.89	8.06 ± 0.93
Fragment^d^*	0.37 ± 0.50	0.32 ± 0.48
Equality^e^*	0.91 ± 0.95	0.87 ± 0.91

*The selected features after performing feature selection are marked by asterisks

*SET* single-embryo transfer, *DET* double-embryo transfer, *IVF* in vitro fertilization, *ICSI* intracytoplasmic sperm injection, *E2* estradiol, *hCG* human chorionic gonadotropin, *MESA* microscopic epididymal sperm aspiration, *TESA* testicular sperm aspiration, *PESA* percutaneous epididymal sperm aspiration

^a^Number of oocytes retrieved; ^b^ for multi-category features, the sum of the proportion for each category may not equal 100% because the missing value exists or another small proportion of category features is not included; ^c^ infertility is encoded by 0 or 1 if the patient is primary or secondary, respectively; ^d^ the fragment is encoded by three values: 1 to 3 representing no fragment, 5–15% fragment, and > 15% fragment, respectively; ^e^ the equality is encoded by five values, 0 to 4, and represent equal, sort of equal, unequal, sort very unequal, and very unequal, respectively.

### Controlled ovarian hyperstimulation, embryo culture, and pregnancy ascertain

IVF patients were treated with controlled ovarian hyperstimulation (COH) by gonadotropin-releasing hormone (GnRH) agonist or GnRH antagonist, as previously described [14, 15]. When two dominant follicles reached 17–18 mm in diameter, oocytes were retrieved transvaginally 36–38 h after a human chorionic gonadotropin (hCG) trigger. Embryos were cultured in G1 medium (Vitrolife, Sweden) after fertilization and transferred on day 3. Subsequently, biochemical pregnancy was ascertained by a positive serum hCG 2 weeks after embryo transfer with serial elevation. Clinical pregnancy was defined as a gestational sac and active fetal heartbeat on ultrasound demonstration 8 weeks after embryo transfer.

### Model construction, feature selection, and validation

**Fig. 1:**
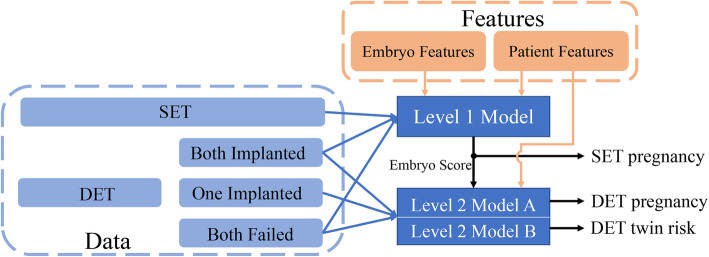
The overall flowchart of the proposed hierarchical model. The first-level model was trained using all data except double embryo transfer (DET), with only one embryo implantation to predict single-embryo implantation outcomes. The second level contains two models, which were trained using DET data to predict both DET implantation outcomes and twin risks

According to previous studies, the outcome of DET is not a simple binomial distribution with respect to two transferred embryos [16]. Therefore, a hierarchical model with two levels was established to learn single embryo implantation potential and the impact of double embryos transferred simultaneously. In the first level, patient and embryo features were applied to predict the implantation chance for single embryo, from which the output value was directly treated as the predicted pregnancy chance. For DET, the respective pregnancy probability of two embryos (P1 and P2) was initially predicted by the first level calculation, then another two features, generated by addition (P1 + P2) and multiplication (P1 × P2), were also included, followed by the combination of generated features and patient features as the input for the second level. Two models were developed in the second level, one to predict the DET pregnancy chance and the other to predict the DET twin risk. The overall flowchart of our model is shown in Fig. 1. The first-level model was trained by all data except DET with only one embryo implantation, whereas the second-level models were trained by all DET data.

The machine learning system applied here was XGBoost [17]. XGBoost is a scalable machine learning system for tree boosting, which uses the boosting technique to train multiple trees. In other words, XGBoost is an ensemble of multiple decision trees. Unlike random forest, which generates trees by randomly selecting a subset of training sets and features, XGBoost generates decision trees one by one, based on the performance of the previous generated trees. And comparing to single decision tree algorithm such as C4.5 or CART, ensemble of trees can achieve better performance. Moreover, this system can automatically deal with missing values and assign the importance score for each feature, which was applied for feature selection. Nineteen features were selected and are marked by asterisks in Table 1.

As the training procedure was completed, the performance of the model was evaluated with the area under the curve (AUC) of the receiver operating characteristic (ROC) curve [18]. Multiple regression analyses were also conducted on the 19 selected features, with the glm function in R [19] to demonstrate the differences between feature importance for prediction and statistical relationship with outcomes.

### Embryo selection strategy development

**Fig. 2:**
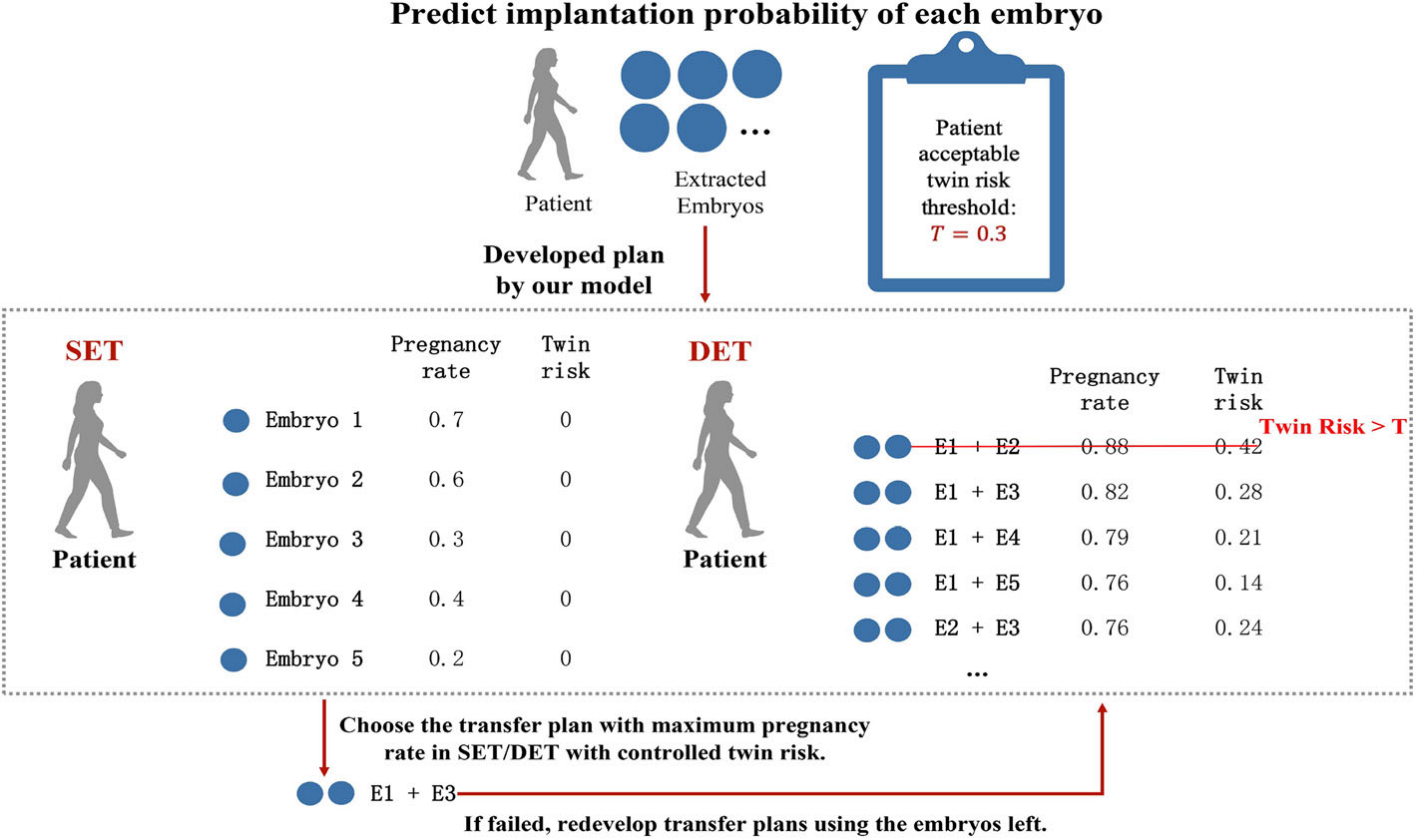
Embryo selection strategy developed by the proposed model. For any given acceptable twin rate threshold, the pregnancy and twin rates could be predicted

Subsequently, an embryo selection strategy to maximize the pregnancy chance with a controlled twin rate threshold setting was developed. As shown in Fig. 2, for any given acceptable twin rate threshold, the pregnancy and twin rate could be predicted for every possible embryo selection strategy, including one or two best embryos to transfer or the combination of one best embryo and one medium-quality embryo. Moreover, if the transfer failed in the fresh cycle, the plan would be redeveloped with the remaining embryos, following the same protocol until all embryos were transferred or a new cycle was started. With this selection strategy, the prediction model could effectively increase the percentages of elective SET patients and provide plausible plans for DET patients in IVF.

### Statistical analysis

Continuous variables were presented as mean ± standard deviation. Categorical variables were presented as percentage encoded by one-hot encoding for analysis. For XGBoost algorithm, we used the implementation from scikit-learn (https://xgboost.readthedocs.io/en/latest/python/ python_api.html) using Python. The parameters were as follows: max_depth = 5, min_child_weight = 1, learning_rate = 0.1, n_estimators = 100, gamma = 0, subsample = 0.8, colsample_bytree = 0.8. Other parameters were set by default.

## Results

### Variable analysis using training data (January 2016 to march 2018)

The results of multiple regression analyses are shown in Table 2. For a SET pregnancy, the following variables remained significant: age, attempts at IVF, estradiol level on hCG day, and endometrial thickness. For DET pregnancy, age, attempts at IVF, endometrial thickness, and the newly added P1 + P2 remained significant. For DET twin risk, age, attempts at IVF, 2 pronucleus (PN)/ metaphase II (M II), and P1 × P2 remained significant.

**Table 2:** Multivariate analysis results of the selected features for SET pregnancy, DET pregnancy and twin risk prediction

Selected features	***P*** value		
	SET	DET	
	Pregnancy	Pregnancy	Twin Risk
Age	0.0222*	< 0.0001*	< 0.0001*
Attempt times of IVF	< 0.001*	< 0.0001*	< 0.0001*
Antral follicle count	0.3332	0.4016	0.4121
Follicle stimulating hormone	0.7307	0.8141	0.4633
Luteinizing hormone	0.3501	0.7230	0.4616
E2 on HCG day	0.0053*	0.9040	0.7684
Endometrial Thickness	0.0046*	< 0.0001*	0.2080
MII	0.9455	0.9444	0.0546
2PN	0.1041	0.9068	0.1021
Oocyte Number	0.7510	0.8897	0.6324
2PN/MII	0.5038	0.7772	0.0148*
Stimulation Protocol			
Agonist Protocol	0.3692	0.2019	0.4961
Endometrial Type			
A	0.8531	0.5324	0.4914
C	0.7138	0.0887	0.4614
Secondary Infertility	0.0816	0.2445	0.6809
Fertilization Method			
ICSI	0.2365	0.5069	0.8492
Embryo Features			
Blastomere Number	0.5422	NU^a^	NU
Fragment	0.1585	NU	NU
Equality	0.5399	NU	NU
Embryo Scores			
P1 + P2	NU	< 0.001*	0.1344
P1 × P2	NU	0.2040	0.007*

*P < 0.05

^a^NU means this feature was not used in the corresponding level

### Feature importance of the hierarchical model

**Fig. 3:**
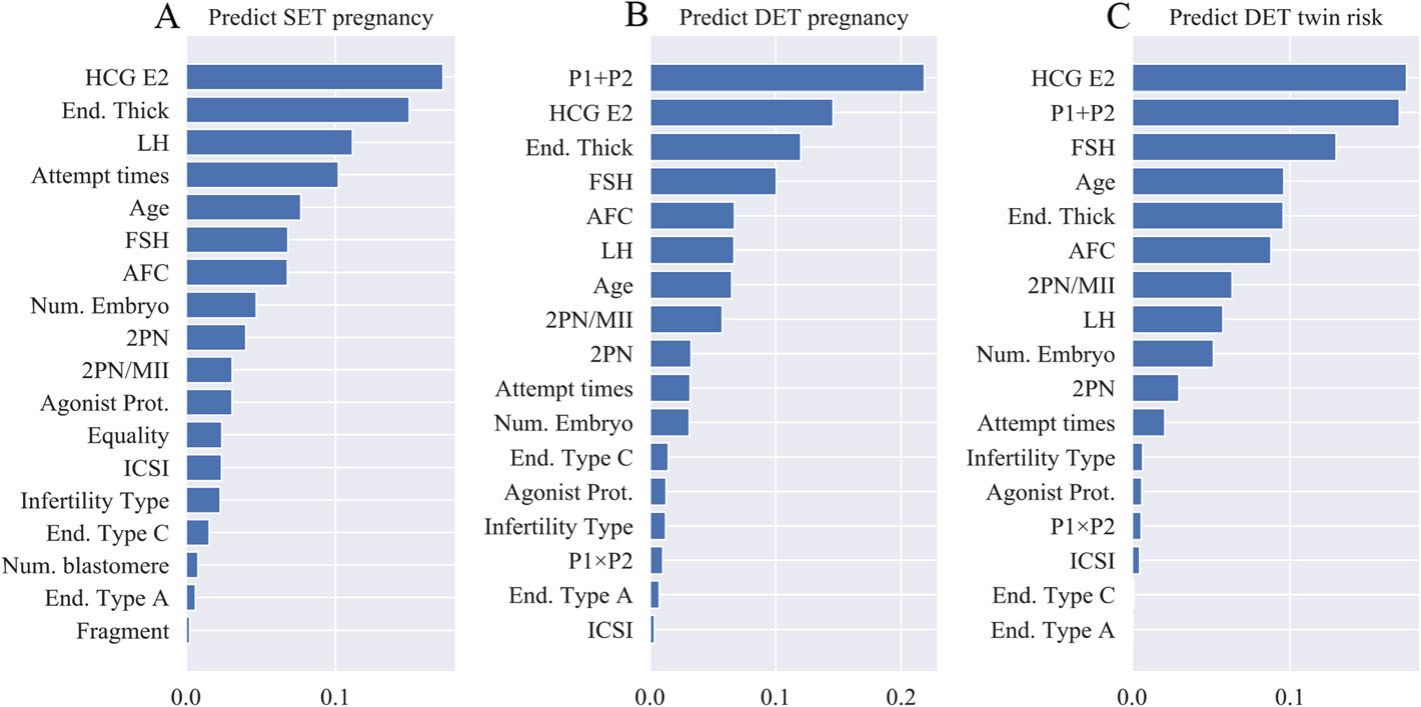
Feature importance in the hierarchical model. Feature importance of (**a**) first-level model for predicting SET pregnancy, (**b**) second-level model for predicting DET pregnancy, and (**c**) second-level model for predicting DET twin risk

In the XGBoost algorithm, the feature importance means the number of times that a feature is used to split the data across all trees. A higher feature importance score represents greater value for prediction. Figure 3 shows the feature importance in the hierarchical model for a SET pregnancy, a DET pregnancy, and DET twin risk. Some features are not shown in Fig. 3 because the corresponding importance was zero, which means these features were not used for prediction.

For SET, as shown in Table 2, the luteinizing hormone (LH) level and follicle-stimulating hormone (FSH) level were not statistically significant, yet they were the third- and sixth-important features for the first-level model. The same phenomenon was observed for FSH, LH, and antral follicle count (AFC) in DET pregnancy and DET twin risk prediction. Although P1 × P2 was significantly correlated with DET twin risk, it was hardly used by either level model.

### Model validation results

**Fig. 4:**
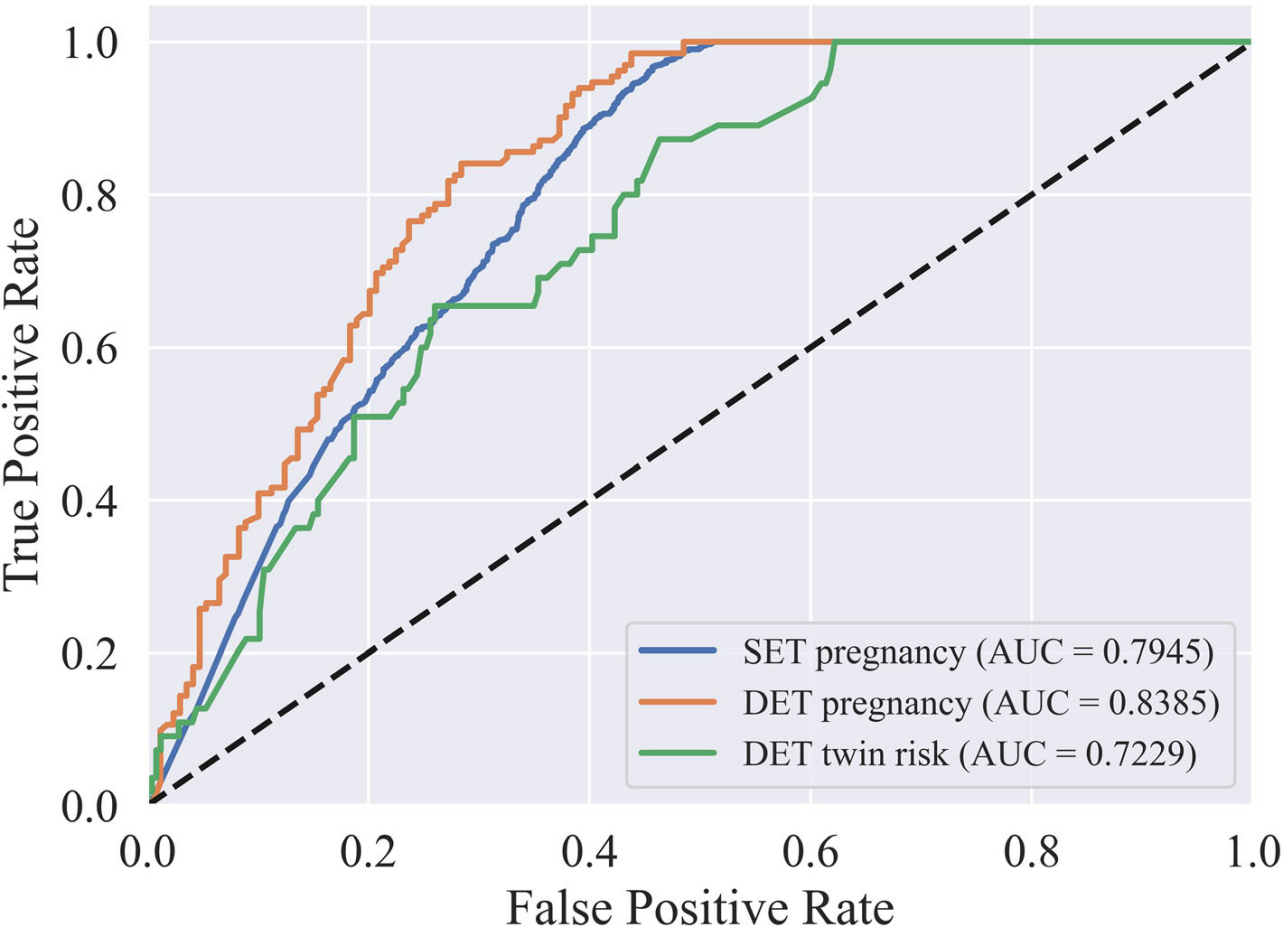
The ROC curve of a single-embryo transfer (SET) pregnancy, double-embryo transfer (DET) pregnancy, and DET twin risk in our method. The average AUCs for a SET pregnancy, a DET pregnancy, and DET twin risk were 0.7945, 0.8385, and 0.7229, respectively

The algorithm was repeated 30 times to eliminate the interference of random factors, and averaged AUCs of 0.7945, 0.8385, and 0.7229 were achieved for SET pregnancy, DET pregnancy, and DET twin risk, respectively. The ROC curve in one single run is shown in Fig. 4.

**Fig. 5:**
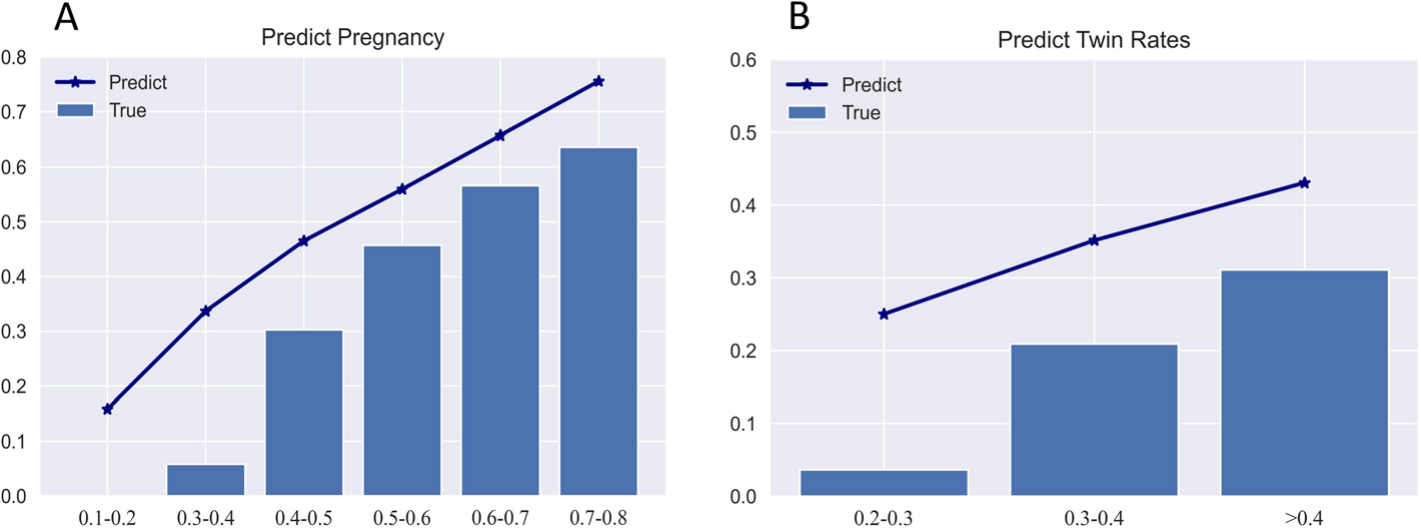
Model validation in 3383 patients in comparing the predicted value with the truly observed percentage. **a** Pregnancy prediction in single-embryo transfer (SET) and double-embryo transfer (DET) versus truly observed percentage. **b** Twin risk prediction in DET versus truly observed twin rate

To validate the effectiveness of the developed embryo selection strategy, the consistency between the predicted chance and the observed actual outcomes was analyzed, as shown in Fig. 5. The trend of predictive and observed rates both in pregnancy and twin risk was basically identical, although with a slight elevation in predictive rates.

**Fig. 6:**
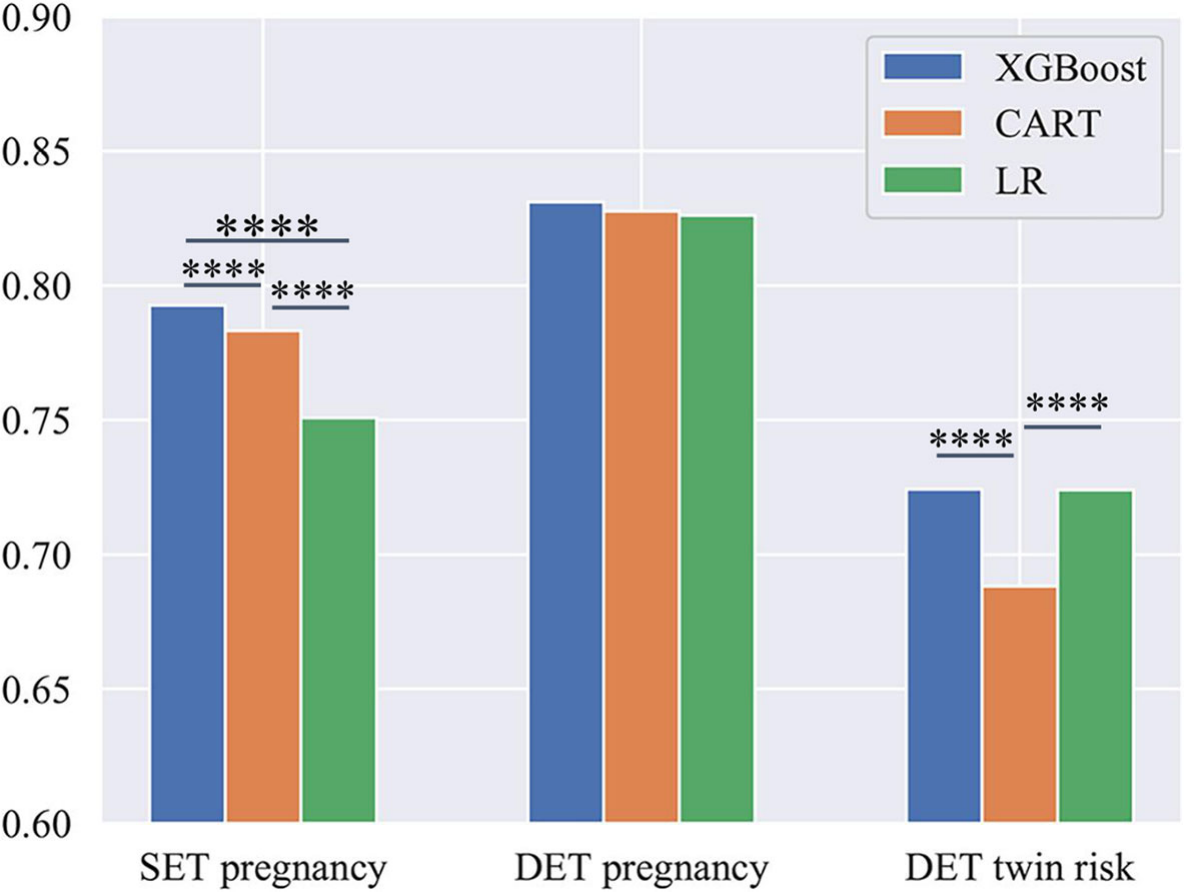
AUC performance comparison among XGBoost, CART, and LR on single-embryo transfer (SET) pregnancy, double-embryo transfer (DET) pregnancy, and DET twin risk prediction. ****P < 0.0001

To validate the prediction performance among XGBoost and other algorithms such as logistic regression (LR) and classification and regression tree (CART), we also performed a non-parametric multiple comparison test using Dunn’s procedure [20], with a *p*-value correction using the False Discovery Rate method [21]. The results are shown in Fig. 6, where the significant ones (*p* < 0.05) are marked in asterisk. In general, XGBoost outperformed the other two algorithms with obvious prediction power in SET pregnancy prediction and DET twin risk prediction and similar performance in DET pregnancy prediction task.

## Discussion

One of the most overwhelming challenges in contemporary assisted reproductive technology (ART) is how to narrow the gap in perinatal and neonatal outcomes between spontaneous pregnancy and assisted pregnancy, usually caused by multiple-embryo transfer and implantation [22]. In recent decades, SET has been advocated in IVF to prevent multiple pregnancies while led to the concerns of decreased IVF success [3, 23, 24]. Therefore, precise embryo selection for SET and twin risk warning for DET are particularly necessary. In this paper, a novel hierarchical model was constructed and validated to optimize embryo selection strategies and successfully predict pregnancy for both SET and DET, as well as predict the twin risk of DET for each individual. Subsequently, we validated our model on 3383 patients, and the results showed that our model had achieved an acceptable performance on embryo selection and twin risk prediction for each individual, as shown in Figs. 4 and 5.

It is generally accepted that embryos with similar morphology present variable implantation probability, depending on other assessed features such as patient characteristics and cycle demographics. Many features, including age, FSH level, anti-Mullerian hormone (AMH) and embryo quality, have been reported as independent impact factors on oocyte variability and embryo implantation potential [5, 25–28]. Machine learning is considered a powerful mathematical tool for correlation analysis when huge data is involved; therefore, AI has been introduced in embryo variability prediction in many articles [29, 30]. By increasing the information of the input features, the predictive power of the proposed model may be improved. Similarly, 19 features from the initial data sets were considered in the current study to construct the predictive power in our model. It is noteworthy that although previous models may have helped predict the implantation potential of a specific embryo, they did not offer a reasonable and optimal guidance to the embryo selection determination in clinical practice.

Minaretzis et al. [31] once presented an embryo selection strategy of transferring one additional good-quality embryo for each 5 years of incremental increase in maternal age to improve IVF outcome by multivariate analysis of factors predictive of successful live birth. Because maternal age was the only influencing factor to be considered, the recommended strategy was not so accurate or comprehensive. Kaufmann et al. [32] reported a neural networks predicting model in IVF, where four input parameters were included and the overall accuracy was 59%. Uyar et al. [33] proposed a Support Vector Machine (SVM) method in embryo implantation prediction in terms of Area Under ROC curve (0.712 ± 0.032), when 12 features were included.

Vaegter et al. [16] also constructed a predictive model for an embryo transfer strategy, in which only one embryo was transferred if the predictive risk of twin implantation was above 15%, using the two highest scored embryos. While validating this predictive model, the actual twin rate was 3.8%, which was far lower than the expected 15% setting, accompanied by a decreased live birth rate. Unlike previous studies, a novel strategy in embryo selection based on an accurate embryo potential prediction and twin risk assessment was developed in our model. The model not only guides a determination of the number of embryos for transfer and the specific embryo selection, but also presents a relatively accurate prediction of the pregnancy rate and twin risk of the corresponding selection scheme.

Because SET was not arbitrarily implemented in all IVF patients, twin pregnancy was inevitable. However, the acceptable twin rate threshold varied among different countries, even among different IVF centers. The greatest strength of our model was that the embryo selection strategy we developed varied correspondingly to patients’ characteristics and embryo morphology parameters as long as different twin rate thresholds were set. Therefore, the selection strategy model is applicable to any IVF center in any country. In such a situation, embryologists can input their accepted twin rate setting on the model to generate their guided embryo selection strategies and present a predictable pregnancy possibility and twin rate assessment.

More important, the predictive twin risk indicated that the transferred two embryos were neither a simplified embryo implantation nor an implantation of two independent samples. This principle was previously discussed in an embryo-uterus modeling framework [34–36]. A higher twin rate than expected would be achieved if the implantation chances of the embryos transferred together were completely independent of one another [16]. As shown in Fig. 3, E2 on hCG day, endometrial thickness, patient age, FSH, and AFC were ranked within top seven in the feature importance for the prediction of SET pregnancy, DET pregnancy, and twin risk, although with slightly different ranking. However, LH and attempt times of IVF, which were key in predicting SET pregnancy, were less weighted in DET pregnancy and twin risk prediction. Considering the variability of confounding factors, a two-level algorithm was introduced in our model for DET prediction to achieve a satisfying predictive model. Furthermore, unlike previous researches, which only considered significant influencing factors in model construction [37–39], concomitant investigation in our DET model provided a novel insight: that even if one variable were not statistically significant, it might still be important for machine learning models to predict final outcomes.

To minimize twin risk, some researchers also constructed a predictable model and validated it in subsequent IVF cycles, and the results showed a significantly reduced twin rate (from 25.2 to 3.8%) [16]. However, the proportion of SETs was greatly increased (11.3 to 75.5%), and surplus embryos would be preserved for future use, resulting in a reduced live birth rate in fresh cycle (29.0 to 25.1%). Even though the cumulative live birth rate (CLBR) was not affected, patients had to pay more fees and spend more time for a successful pregnancy in subsequent frozen-thawed cycles. Luke et al. [40] also reported a similar conclusion of comparative CLBR and decreased multiple births in SETs over two cycles, compared to DET in one cycle. In our algorithm, conversely, the pregnancy rate and twin risk were assessed and predicted simultaneously in the fresh cycle, and further validation showed that it effectively reduced twin risk without compromising clinical pregnancy in the fresh cycle.

The recommended embryo selection strategy in our model provides decision support to embryologists with higher accuracy and efficiency. For a large proportion of IVF patients with suboptimal prognosis, which plan to choose was a dilemma to embryologists, because prediction judgment was based on their clinical experience instead of on the analysis of thousands of embryos and patient records prior to each embryo transfer [41, 42]. Besides, it may also act as a counseling tool for clinicians to evaluate the chance of pregnancy before the transfer procedure.

Previous research to establish similar predictive models were problematic due to the limited number of involved features or dramatic distinction between the predicted value and the actual situation [16, 31]. The model in the current study guides a determination of the number of embryos for transfer and the specific embryo selection, based on the analysis of a large number of features. In addition, the predicted pregnancy rate and twin risk of the corresponding selection scheme were relatively more accurate. More significantly, our model provides a flexible strategy, with individualized embryo selection for any given patient and varied operations corresponding with any setting acceptable twin rate threshold.

One shortcoming of our predictive model was that the predicted mean value was slightly elevated compared to the actual observed rate for both pregnancy and twin rates in validation, although a modest discrimination was also reported in the previous prediction model in ART [43–45]. The model should be adjusted and improved to make the predictive value more closely agree with the actual virtue in the future verification. Besides, the model was developed and validated using data from a single center. As future work, we will need to be much more rigorous with additional data sets demonstrating the predictive value, using the same parameters, as well as applying them in different centers.

## Conclusions

In our study, we constructed and validated an individualized embryo selection strategy and pregnancy prediction model developed by stacking machine learning. This prediction model could provide an accurate and individualized embryo selection strategy for any given patient as well as in any twin risk threshold setting and balance a delicate correlation between clinical pregnancy and twin risk rate; it therefore promises a better IVF outcome without compromising pregnancy success and controlled twin risk.

## Data Availability

All data generated or analyzed during this study are included in this published article.

## Abbreviations

IVF: In vitro fertilization
SET: Single embryo transfer
DET: Double embryo transfer
AI: Artificial intelligence
COH: Controlled ovarian hyperstimulation
GnRH: Gonadotropin-releasing hormone
hCG: Human chorionic gonadotropin
AUC: Area under the curve
ROC: Receiver operating characteristic
FSH: Follicle-stimulating hormone
LH: Luteinizing hormone
AFC: Antral follicle count
LR: Logistic regression
CART: Classification and regression tree
ART: Assisted reproductive technology
AMH: Anti-Mullerian hormone
CLBR: Cumulative live birth rate
